# Author Correction: Demonstration of Shor’s factoring algorithm for N = 21 on IBM quantum processors

**DOI:** 10.1038/s41598-023-28616-x

**Published:** 2023-01-24

**Authors:** Unathi Skosana, Mark Tame

**Affiliations:** Department of Physics, Stellenbosch University, Matieland, 7602 South Africa

Correction to: *Scientific Reports* https://doi.org/10.1038/s41598-021-95973-w, published online 16 August 2021

The original version of this Article contained errors in Figure 7, where the order of the outcome bits was reversed.

The peak outcomes should be 011 and 101, corresponding to the integers 3 and 5. Both of these integers give the correct period r = 3 when continued fractions are performed, leading to the successful factorization of N = 21. In the original version of the paper only the integer 5 was identified and shown to state the correct period. The original Figure 7 and accompanying legend appear below.

**Figure 7:**
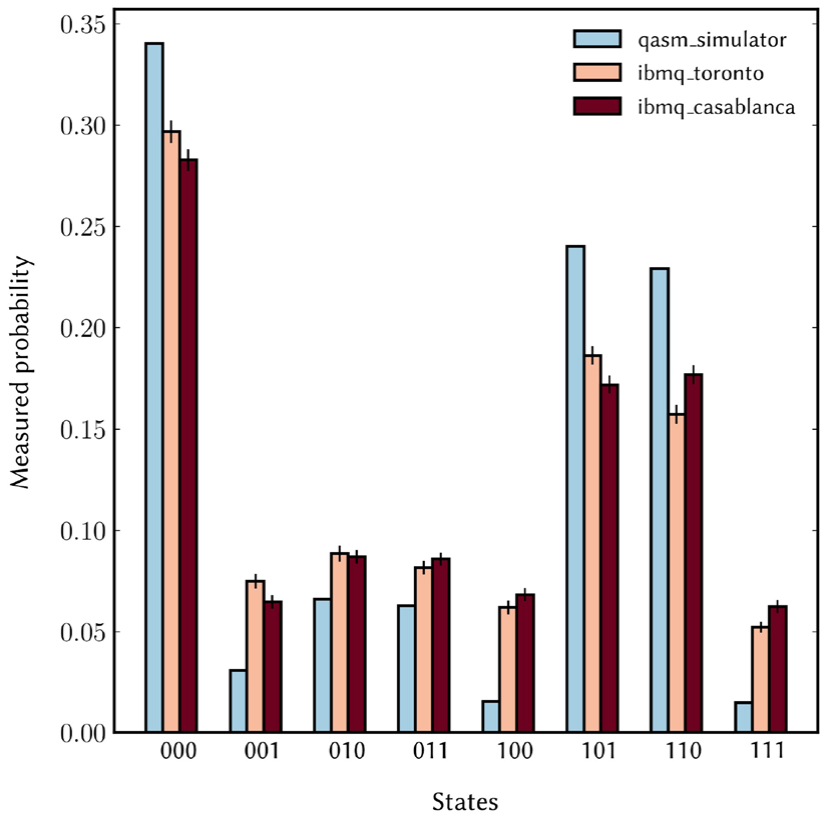
Results of the complete quantum order-finding routine for *N* = 21 and *a* = 4. On each processor, the circuit was executed 8192 × 100 times with measurement error mitigation. The error bars represent 95% confidence intervals around the mean value of each histogram bin (see Supplementary information IV). The simulator probabilities show the ideal case.

Additionally, in the Compiled Shor’s algorithm section, under the subheading ‘Modular exponentiation’ the mappings for the unitary operation $\hat{U}^{2}$ were incorrect.

“Similarly, the implementation of $\hat{U}^{2}$ can be simplified by noting that the states $|1\rangle$ and $|4\rangle$ are the only non-zero amplitude states in the work register after $\hat{U}^{1}$ may have been applied, thus prompting us to only consider $|1\rangle \mapsto |4\rangle$ and $|4\rangle \mapsto |16\rangle$.”

now reads:

“Similarly, the implementation of $\hat{U}^{2}$ can be simplified by noting that the states $|1\rangle$ and $|4\rangle$ are the only non-zero amplitude states in the work register after $\hat{U}^{1}$ may have been applied, thus prompting us to only consider $|1\rangle \mapsto |16\rangle$ and $|4\rangle \mapsto |1\rangle$.”

Furthermore, in the Experiments section, under the subheading ‘Performance’ the probability of the outcomes on the two processors was quoted incorrectly with respect to the data shown in Figure 7.

“The outcomes $|101\rangle$ and $|110\rangle$ occur with probability ∼14% and ∼16% respectively.”

now reads:

“The outcomes $|011\rangle$ and $|101\rangle$ occur with probability ∼16% and ∼19% on ibmq_toronto and ∼18% and ∼17% on ibmq_casablanca, respectively.”

As a result, in the Supplementary Information, under the subheading ‘VII. CONTINUED FRACTIONS AND CONVERGENTS’

“Consider the following example of the final measurement outcomes from Fig. 7 in the main text, where the outcomes $|110\rangle = |6\rangle |$ and $|101\rangle = |5\rangle$ are peaked in the outcome distribution and we have used the integer representation of the binary outcome.”

now reads:

“Consider the following example of the final measurement outcomes from Fig. 7 in the main text, where the outcome $|110\rangle = |6\rangle$ is not a peak but $|101\rangle = |5\rangle |$ is a peak in the outcome distribution and we have used the integer representation of the binary outcomes.”

Finally, under the same subheading,

“Looking at the former and latter computed convergents, we note that the third convergent of the latter correctly gives r′ = 3 while the convergents of the former do not give the correct order when tested using ar′ mod N = 1.”

now reads:

“Looking at the former and latter computed convergents, we note that the fourth convergent of the latter correctly gives r′ = 3 while the convergents of the former do not give the correct order when tested using ar′ mod N = 1. The same process can be applied to the outcome |011⟩ = |3⟩, which is a peak and correctly gives r′ = 3.”

The original Supplementary Information file is provided below.

The original Article and accompanying Supplementary Information file have been corrected

## Supplementary Information

Supplementary Information.

