# Author Correction: Demonstration of Shor’s factoring algorithm for N = 21 on IBM quantum processors

**DOI:** 10.1038/s41598-023-30980-7

**Published:** 2023-03-14

**Authors:** Unathi Skosana, Mark Tame

**Affiliations:** Department of Physics, Stellenbosch University, Matieland, 7602 South Africa

Correction to: *Scientific Reports* 10.1038/s41598-021-95973-w, published online 16 august 2021

The original version of this Article contained errors.

In the Experiments section, under the subheading ‘Performance’,

“However, the amplification of the peaks |000⟩, |101⟩ and |110⟩ is clearly visible from the processor outcomes”.

now reads:

“However, the amplification of the peaks |000⟩, |011⟩ and |101⟩ is clearly visible from the processor outcomes.”

In addition, under the subheading ‘Factoring N = 21’,

“The measured probability distributions in Fig. 7 are peaked in probability for the outcomes 000 (ϕs = 0), 101 (ϕs = 5), and 110 (ϕs = 6), with ideal probabilities of 0.35, 0.25 and 0.25, respectively.”

now reads:

“The measured probability distributions in Fig. 7 are peaked in probability for the outcomes 000 (ϕs = 0), 011 (ϕs = 3), and 101 (ϕs = 5), with ideal probabilities of 0.35, 0.25 and 0.25, respectively.”

Finally, under the same subheading,

“On the other hand, the continued fraction expansion of ϕ = 6/8 gives {0,1,3/4} and incorrectly gives r = 4 as the order (see Supplementary information VII for details).”

now reads:

“On the other hand, the continued fraction expansion of ϕ = 3/8 also gives r = 3, while adjacent outcomes that have an appreciable but lower probability do not give the correct order, for example ϕ = 6/8 gives {0,1,3/4} and incorrectly gives r = 4 as the order (see Supplementary information VII for details).”

The original Article has been corrected.

