# Supplementary material for: Demonstration of Shor’s factoring algorithm for N = 21 on IBM quantum processors
Source: Sci Rep. 2021 Aug 16;11:16599. doi: 10.1038/s41598-021-95973-w (PMC8368060; doi:10.1038/s41598-021-95973-w)
Supplement: Supplementary file 1 — Supplementary Information. [file 41598_2021_95973_MOESM1_ESM.pdf]

Supplementary information for
Demonstration of Shor's factoring algorithm for N=21 on IBM quantum processors

Unathi Skosana* and Mark Tame
Department of Physics, Stellenbosch University, Matieland 7602, South Africa

(Dated: June 17, 2021)

I. POSTSELECTION SCALING

In order to do mid-circuit measurements and post select the outcomes, we need to know the basis to measure in for each of the qubits. Thus, one would need to measure qubit 1 (or the first iteration in the recycling case) in the $\{|+\rangle, |-\rangle\}$ basis, then qubit 2 (or the second iteration in the recycling case) in either the $\{S|+\rangle, S|-\rangle\}$ or $\{|+\rangle, |-\rangle\}$ basis, then qubit 3 in either the $\{TS|+\rangle, TS|-\rangle\}$, $\{S|+\rangle, S|-\rangle\}$ or $\{|+\rangle, |-\rangle\}$ basis. Thus, the number of measurements needed scales as $n!$, which grows faster than an exponential with constant base, e.g. 2^n. So in general the speed up gained would be lost for general factoring using a post selection method, *i.e.* factoring numbers larger than 21.

II. EFFECT OF RELATIVE PHASE TOFFOLIS

Below we show the compiled circuit for the period-finding routine and label specific instances during the evolution of the computation. The aim is to show the invariance of the computation when replacing Toffoli gates with relative phase Toffoli gates that use fewer resources.

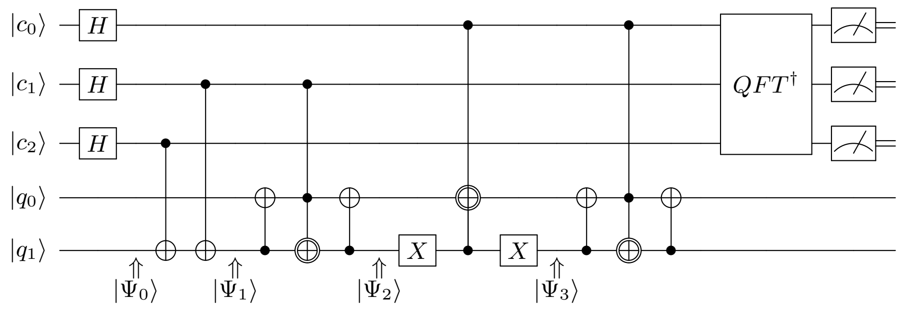

FIG. 1. States in both registers at various points during the execution of the circuit.

* ukskosana@gmail.com

The states are various points of the evolution are given explicitly as

$$\begin{aligned}
|\Psi_0\rangle &= |+\rangle_{c_0} |+\rangle_{c_1} |+\rangle_{c_2} |0\rangle_{q_0} |0\rangle_{q_1} , \\
|\Psi_1\rangle &= |+\rangle_{c_0} (|0\rangle_{c_1} |0\rangle_{c_2} |0\rangle_{q_0} |0\rangle_{q_1} + |0\rangle_{c_1} |1\rangle_{c_2} |0\rangle_{q_0} |1\rangle_{q_1} + \\
&\qquad |1\rangle_{c_1} |0\rangle_{c_2} |0\rangle_{q_0} |1\rangle_{q_1} + |1\rangle_{c_1} |1\rangle_{c_2} |0\rangle_{q_0} |0\rangle_{q_1}), \\
|\Psi_2\rangle &= |0\rangle_{c_0} |0\rangle_{c_1} |0\rangle_{c_2} |0\rangle_{q_0} |0\rangle_{q_1} + |0\rangle_{c_0} |0\rangle_{c_1} |1\rangle_{c_2} |0\rangle_{q_0} |1\rangle_{q_1} + \\
&\quad |0\rangle_{c_0} |1\rangle_{c_1} |0\rangle_{c_2} |1\rangle_{q_0} |0\rangle_{q_1} + |0\rangle_{c_0} |1\rangle_{c_1} |1\rangle_{c_2} |0\rangle_{q_0} |0\rangle_{q_1} + \\
&\quad |1\rangle_{c_0} |0\rangle_{c_1} |0\rangle_{c_2} |0\rangle_{q_0} |0\rangle_{q_1} + |1\rangle_{c_0} |0\rangle_{c_1} |1\rangle_{c_2} |0\rangle_{q_0} |1\rangle_{q_1} + \\
&\quad |1\rangle_{c_0} |1\rangle_{c_1} |0\rangle_{c_2} |1\rangle_{q_0} |0\rangle_{q_1} + |1\rangle_{c_0} |1\rangle_{c_1} |1\rangle_{c_2} |0\rangle_{q_0} |0\rangle_{q_1} , \\
|\Psi_3\rangle &= |0\rangle_{c_0} |0\rangle_{c_1} |0\rangle_{c_2} |0\rangle_{q_0} |0\rangle_{q_1} + |0\rangle_{c_0} |0\rangle_{c_1} |1\rangle_{c_2} |0\rangle_{q_0} |1\rangle_{q_1} + \\
&\quad |0\rangle_{c_0} |1\rangle_{c_1} |0\rangle_{c_2} |1\rangle_{q_0} |0\rangle_{q_1} + |0\rangle_{c_0} |1\rangle_{c_1} |1\rangle_{c_2} |0\rangle_{q_0} |0\rangle_{q_1} + \\
&\quad |1\rangle_{c_0} |0\rangle_{c_1} |0\rangle_{c_2} |1\rangle_{q_0} |0\rangle_{q_1} + |1\rangle_{c_0} |0\rangle_{c_1} |1\rangle_{c_2} |0\rangle_{q_0} |1\rangle_{q_1} + \\
&\quad |1\rangle_{c_0} |1\rangle_{c_1} |0\rangle_{c_2} |0\rangle_{q_0} |0\rangle_{q_1} + |1\rangle_{c_0} |1\rangle_{c_1} |1\rangle_{c_2} |1\rangle_{q_0} |0\rangle_{q_1} .
\end{aligned} \tag{1}$$

Looking at the state $|\Psi_1\rangle$, one can see that none of its constituent states is transformed into $|1\rangle_{c_1} |0\rangle_{q_0} |1\rangle_{q_1}$ by the CX gate that follows, since the state $|1\rangle_{c_1} |1\rangle_{q_0} |1\rangle_{q_1}$ that would be transformed to the former is not present in $|\Psi_1\rangle$. Thus the relative phase Toffoli gate does not affect the phase in the registers.

Similarly for $|\Psi_2\rangle$, the state $|1\rangle_{c_0} |1\rangle_{q_0} |0\rangle_{q_1}$ is not present when the subsequent relative phase Toffoli gate is applied because the state $|1\rangle_{c_0} |1\rangle_{q_0} |1\rangle_{q_1}$ is absent from the register for $|\Psi_2\rangle$ and this is needed when $\hat{X}$ is applied to qubit q_1.

The scenario for $|\Psi_3\rangle$ is the same as that of $|\Psi_1\rangle$, the only difference is the control is now c_0.

The Margolus gates in this particular quantum circuit never encounter the basis state $|101\rangle$, thus the operation of the circuit remains unchanged by the replacement of full Toffoli gates with their respective relative phase counterparts.

III. IBM QUANTUM EXPERIENCE

The experiments in this paper were conducted on the IBM Quantum Experience **ibmq_toronto** and **ibmq_casablanca** processors through the software development kit Qiskit [1]. Each experiment reported here was conducted on the date shown in the table below.

Experiment	Date
Compiled quantum order-finding on **ibmq_casablanca**	2020/12/03
State tomography on **ibmq_casablanca**	2020/12/04
Verification of entanglement on **ibmq_casablanca**	2020/12/04
Compiled quantum order-finding on **ibmq_toronto**	2020/12/06
Verification of entanglement on **ibmq_toronto**	2020/12/07
State tomography on **ibmq_toronto**	2020/12/16

TABLE I. Dates of experiments.

For characterization purposes, the compiled quantum order-finding experiments were submitted in batches of 900 circuits with each circuit having 8192 measurement shots. In total, 900×8192 measurements were made. In choosing the qubit device mappings shown in the main paper, preference was given to the qubit pairs with relatively small CX error rates. Tables II and III show reported single qubit-error rates for **ibmq_toronto** and **ibmq_casablanca** respectively, where $U2(\phi, \lambda) = R_z(\phi)R_y(\frac{\pi}{2})R_z(\lambda)$. Table IV shows the CX error rates for the two processors. The dates of the experiments are given in the captions.

Qiskit's state tomography fitter uses a least-squares fitting to find the closest density matrix described by Pauli measurement results [2]. On an n-qubit system, the fitter requires measurement results from executing 3^n circuits. This makes state tomography on large circuits in impractical. Thus only 30 state tomography experiments were performed for the three control register qubits and in total $3^3 \times 30 \times 8192$ measurement were made.

	$U2$ gate error rate	Readout error rate
Q0	6.010×10^{-2}	4.39×10^{-4}
Q1	3.14×10^{-2}	2.12×10^{-4}
Q2	2.98×10^{-2}	1.96×10^{-4}
Q3	9.30×10^{-3}	5.74×10^{-4}
Q4	1.34×10^{-2}	2.097×10^{-4}

TABLE II. Reported single-qubit gate errors on 16 December 2020.

	$U2$ gate error rate	Readout error rate
Q0	2.16×10^{-2}	2.18×10^{-4}
Q1	1.31×10^{-2}	4.042×10^{-4}
Q2	1.54×10^{-2}	2.78×10^{-4}
Q3	9.30×10^{-2}	2.62×10^{-4}
Q4	1.67×10^{-2}	4.96×10^{-4}

TABLE III. Reported single-qubit gate errors on 06 December 2020.

In reducing the effect of noise due to final measurement errors, Qiskit recommends a measurement error mitigation approach. The approach starts off by creating circuits that each perform a measurement of the 2^n basis states. The measurement counts of the 2^n basis state measurements are put into a column vector C_{noisy}, arranged in ascending order by the value of their measurement bitstring, *i.e.* $00\ldots00$ is the first element, the next is $00\ldots01$ and so on. The approach assumes that there is a matrix M called the calibration matrix, such that

$$C_{\text{noisy}} = MC_{\text{ideal}}, \tag{2}$$

where C_{ideal} is a column vector of measurement counts in the absence of noise. If M is invertible then, then C_{noisy} can transformed into C_{ideal} by finding M^{-1}

$$C_{\text{ideal}} = M^{-1}C_{\text{noisy}}. \tag{3}$$

Qiskit [3] uses a least-squares fit to calculate an approximate M^{-1} by some other matrix $\tilde{M}^{-1}$, as in general M is not invertible, giving

$$C_{\text{mitigated}} = \tilde{M}^{-1}C_{\text{noisy}}. \tag{4}$$

The entries of the column vector $C_{\text{mitigated}}$ correspond to the mitigated measurement counts in same order as before. The entirety of the results reported in our work make of use of this approach.

IV. ERROR BARS

All the confidence intervals of the data presented here were established via non-parametric bootstrap resampling techniques. In order to place the constraint that the measurement counts should sum to the number of experimental shots, a sample contains data as column vectors of outcomes of some experiment. In each round, the resampling draws entire column vectors whose elements respect the aforementioned constraint. For each outcome across the column vectors, mean estimates are obtained and a confidence interval around the estimates can be appropriately constructed.

To elucidate the above, consider the following example. Consider the outcomes of a two-qubit experiment with experimental shots of 8192 repeated 4 times, as shown in Table V below.

	**ibmq_toronto**	**ibmq_casablanca**
CX (0,1)	6.620×10^{-3}	9.126×10^{-3}
CX (1,4)	8.214×10^{-3}	1.114×10^{-2}
CX (2,1)	7.152×10^{-3}	7.446×10^{-3}
CX (3,2)	6.824×10^{-3}	1.337×10^{-2}

TABLE IV. Reported CX gate errors on 06 December (**ibmq_casablanca**) and 16 December (**ibmq_toronto**) 2020.

Outcomes	Counts			
	Exp. 1	Exp. 2	Exp. 3	Exp. 4
00	2335	2208	2406	2203
01	665	690	633	656
10	183	100	197	177
11	5009	5192	4956	5156

TABLE V. Example data for a two-qubit experiment repeated 4 times for illustrating how bootstrap resampling was done.

Suppose we resampled the experiments $1, 1, 2, 4$ from Table V, making a bootstrap sample of size 5.

$$\begin{aligned} B = [&[2335, 665, 183, 5009], \\ &[2335, 665, 183, 5009], \\ &[2208, 690, 100, 5192], \\ &[2203, 656, 177, 5156]]. \end{aligned} \tag{5}$$

From this, we can obtain appropriately the bootstrap sample for each outcome (corresponding to an index), *e.g.* the bootstrap sample for the outcomes at index 0 (outcome 00) is

$$B_0 = [2335, 2335, 2208, 2203]. \tag{6}$$

The bootstrap mean estimates and confidence intervals can then be performed for each outcome while respecting the constraint of the measurement counts summing up to the total number of experimental shots.

V. PAULI MEASUREMENTS

As an example, consider the measurement of the Pauli expectation value $\langle ZZZZZ \rangle$. Let p_{ijklm} denote the probability for a computational basis measurement $\{|0\rangle, |1\rangle\}$ of five qubits to output the binary string $ijklm$, *i.e.* p_{00000} denotes the probability to measure all the qubits in $|0\rangle$ state. To calculate $\langle ZZZZZ \rangle$ we can combine these probabilities as given in the equation below

$$\begin{aligned} \langle ZZZZZ \rangle = p_{00000} &- p_{00010} - p_{00100} + p_{00101} + p_{00110} - p_{01000} + p_{01001} + p_{01010} + p_{01100} - p_{01101} - \\ p_{01110} &+ p_{01111} - p_{10000} + p_{10001} + p_{10010} - p_{10011} + p_{10100} - p_{10101} - p_{10110} + p_{10111} + \\ p_{11000} &- p_{11001} - p_{11010} + p_{11011} - p_{11100} + p_{11101} + p_{11110} - p_{11111}. \end{aligned} \tag{7}$$

Similarly, the expectation $\langle IZIZI \rangle$ is given by

$$\begin{aligned} \langle IZIZI \rangle = p_{00000} &- p_{00010} + p_{00100} + p_{00101} - p_{00110} - p_{01000} - p_{01001} + p_{01010} - p_{01100} - p_{01101} + \\ p_{01110} &+ p_{01111} + p_{10000} + p_{10001} - p_{10010} - p_{10011} + p_{10100} + p_{10101} - p_{10110} - p_{10111} - \\ p_{11000} &- p_{11001} + p_{11010} + p_{11011} - p_{11100} - p_{11101} + p_{11110} + p_{11111}. \end{aligned} \tag{8}$$

However, the terms in the equation above are given by the marginalization of the distribution measured in Eq. (7) across the outcome space of qubits 1, 3 and 5. By considering all such marginalizations of the distribution in Eq. (7), we obtain the set of Pauli expectation values that can be derived from a measurement of $\langle ZZZZZ \rangle$, namely

$$\begin{aligned} \{ &ZZZZI, ZZZIZ, ZZZII, ZZIZZ, ZZIZI, ZZIIZ, ZZIII, ZIZZZ, ZIZZI, ZIZIZ, ZIZII, ZIIZZ, \\ &ZIIZI, ZIIIZ, ZIIII, IZZZZ, IZZZI, IZZIZ, IZZII, IZIZZ, IZIZI, IZIIZ, IZIII, IIZZZ, \\ &IIZZI, IIZIZ, IIZII, IIIZZ, IIIZI, IIIIZ \}. \end{aligned} \tag{9}$$

After applying what is described above to the Pauli decomposition of the ideal state $\rho = |\Psi\rangle\langle\Psi|$, we reduce the number of terms that we need to measure from 293 to 79 terms, as given below

$$
\begin{aligned}
\{\, & XXXXZ, XXXZX, XXXZZ, XXYYZ, XXYZY, XXZXX, XXZXZ, XXZYY, XXZZX, XYXYZ, \\
& XYXZY, XYYXZ, XYYZX, XYYZZ, XYZXY, XYZYX, XYZYZ, XYZZY, XZXXX, XZXYY, \\
& XZXZZ, XZYXY, XZYYX, XZZXZ, XZZZX, YXXYZ, YXXZY, YXYXZ, YXYZX, YXYZZ, \\
& YXZXY, YXZYX, YXZYZ, YXZZY, YYXXZ, YYXZX, YYXZZ, YYYYZ, YYYZY, YYZXX, \\
& YYZXZ, YYZYY, YYZZX, YZXXY, YZXYX, YZYXX, YZYYY, YZYZZ, YZZYZ, YZZZY, \\
& ZXXXZ, ZXXZX, ZXXZZ, ZXYYZ, ZXYZY, ZXZXX, ZXZXZ, ZXZYY, ZXZZX, ZYXYZ, \\
& ZYXZY, ZYYXZ, ZYYZX, ZYYZZ, ZYZXY, ZYZYX, ZYZYZ, ZYZZY, ZZXXX, ZZXXZ, \\
& ZZXYY, ZZXZX, ZZYXY, ZZYYX, ZZYYZ, ZZYZY, ZZZXX, ZZZYY, ZZZZZ \,\}.
\end{aligned}
\tag{10}
$$

VI. MAXIMUM OVERLAP WITH RESPECT TO THE BIPARTITIONS

The values listed below were obtained using the software package QUBIT4MATLAB [4]. Here, $|\phi\rangle$ is a pure biseparable state in some defined bipartite partition (bipartition), *i.e.* an unentangled product state with respect to this bipartition, and $|\Psi\rangle$ is the ideal state in both the control and work registers preceding the application of the QFT to the control register.

$$
\begin{aligned}
\max_{\phi\in\{(c_1)(c_0c_2q_0q_1)\}} |\langle\phi|\Psi\rangle|^2 &= 0.500, \\
\max_{\phi\in\{(c_2)(c_0c_1q_0q_1)\}} |\langle\phi|\Psi\rangle|^2 &= 0.500, \\
\max_{\phi\in\{(q_0)(c_0c_1c_2q_1)\}} |\langle\phi|\Psi\rangle|^2 &= 0.750, \\
\max_{\phi\in\{(q_1)(c_0c_1c_2q_0)\}} |\langle\phi|\Psi\rangle|^2 &= 0.625, \\
\max_{\phi\in\{(c_0c_1)(c_2q_0q_1)\}} |\langle\phi|\Psi\rangle|^2 &= 0.500, \\
\max_{\phi\in\{(c_0c_2)(c_1q_0q_1)\}} |\langle\phi|\Psi\rangle|^2 &= 0.500, \\
\max_{\phi\in\{(c_0q_0)(c_1c_2q_1)\}} |\langle\phi|\Psi\rangle|^2 &= 0.427, \\
\max_{\phi\in\{(c_0q_1)(c_1c_2q_0)\}} |\langle\phi|\Psi\rangle|^2 &= 0.570, \\
\max_{\phi\in\{(q_0q_1)(c_0c_1c_2)\}} |\langle\phi|\Psi\rangle|^2 &= 0.375, \\
\max_{\phi\in\{(c_0q_1)(c_0c_1q_0)\}} |\langle\phi|\Psi\rangle|^2 &= 0.570, \\
\max_{\phi\in\{(c_1q_1)(c_0c_2q_0)\}} |\langle\phi|\Psi\rangle|^2 &= 0.570, \\
\max_{\phi\in\{(c_1q_0)(c_0c_2q_1)\}} |\langle\phi|\Psi\rangle|^2 &= 0.427, \\
\max_{\phi\in\{(c_2q_0)(c_0c_1q_1)\}} |\langle\phi|\Psi\rangle|^2 &= 0.427, \\
\max_{\phi\in\{(c_1c_2)(c_0q_0q_1)\}} |\langle\phi|\Psi\rangle|^2 &= 0.500.
\end{aligned}
\tag{11}
$$

For a given separation of the qubits into two partitions (a bipartition), *e.g.* $(c_1)(c_0c_2q_0q_1)$, there is a pure product state $|\phi\rangle$ with respect to these partitions, *i.e.* no entanglement between the partitions, that maximizes the overlap squared with the ideal state. The value of the overlap squared between this product state and the ideal state, *e.g.* 0.5, is therefore the highest value that can be obtained for an unentangled state between the partitions. Thus, if a given state has an overlap squared larger than 0.5 it must be an entangled state with respect to the partitions. The value of the maximum overlap squared changes for the different partitions chosen as it depends on the structure of the ideal state. The above results extend to mixed states across the bipartitions due to the convex sum nature of quantum states [4].

VII. CONTINUED FRACTIONS AND CONVERGENTS

A $2L+1$ bit rational number φ is said to have a continued fraction expansion if it can be written as

$$\varphi \equiv [a_0, a_1, \ldots, a_n] \equiv a_0 + \cfrac{1}{a_1 + \frac{1}{a_2 + \frac{1}{\cdots + \frac{1}{a_n}}}}, \tag{12}$$

where n is a finite integer and the a_i's are integers. Additionally, if $\varphi < 1$, we have $a_0 = 0$. The convergents of the continued fraction expansion are the rationals,

$$a_0, a_0 + \frac{1}{a_1}, a_0 + \frac{1}{a_1 + \frac{1}{a_2}}, \cdots \tag{13}$$

If a rational number s/r satisfies the following inequality

$$\left|\frac{s}{r} - \varphi\right| \leq \frac{1}{2r^2}, \tag{14}$$

then s/r will appear as a convergent in the continued fraction expansion of φ. If φ is an approximation of s/r accurate to $2L+1$ bits, then we have $|s/r - \varphi| \leq 1/2^{2L+1}$. For $r \leq N \leq 2^L$, we have that $1/2^{2L+1} \leq 1/2r^2$. Therefore, since the inequality holds for the approximation φ, there is a classical algorithm that can compute the convergents of φ, and produce integers s', r' such that $\gcd(s', r') = 1$ in $\mathcal{O}(L^3)$ operations [5]. We can then check if r' is the order of a and N by testing whether $a^{r'} \bmod N = 1$. Note that in our approach, $\varphi = \varphi_s/2^n \simeq s/r$ is not an approximation that is accurate to $2L+1$ bits as above, but is a further approximation of s/r depending on the resolution, *i.e.* the number of iterations, or alternatively qubits in the control register.

Consider the following example of the final measurement outcomes from Fig. 7 in the main text, where the outcomes $|110\rangle = |6\rangle$ and $|101\rangle = |5\rangle$ are peaked in the outcome distribution and we have used the integer representation of the binary outcome. The former outcome gives $\varphi = \frac{6}{2^3}$ and latter gives $\varphi = \frac{5}{2^3}$. Computing the continued fractions of the former gives

$$\begin{aligned} \frac{6}{8} &= \frac{3}{4}, \\ \frac{3}{4} &= 0 + \frac{1}{\frac{4}{3}}, \\ \frac{3}{4} &= 0 + \frac{1}{1 + \frac{1}{3}}. \end{aligned} \tag{15}$$

Thus

$$\frac{6}{8} = [0, 1, 3]. \tag{16}$$

Computing the convergents according to Eq. (13) gives $0, 1, 3/4$.

On the other hand, computing the continued fractions of the latter φ gives

$$\begin{aligned} \frac{5}{8} &= 0 + \frac{1}{\frac{8}{5}}, \\ \frac{5}{8} &= 0 + \frac{1}{1 + \frac{3}{5}}, \\ \frac{5}{8} &= 0 + \frac{1}{1 + \frac{1}{\frac{5}{3}}}, \\ \frac{5}{8} &= 0 + \frac{1}{1 + \frac{1}{1 + \frac{2}{3}}}, \\ \frac{5}{8} &= 0 + \frac{1}{1 + \frac{1}{1 + \frac{1}{\frac{3}{2}}}}, \\ \frac{5}{8} &= 0 + \frac{1}{1 + \frac{1}{1 + \frac{1}{1 + \frac{1}{2}}}}. \end{aligned} \tag{17}$$

This gives

$$\frac{5}{8} = [0, 1, 1, 1, 2]. \tag{18}$$

Computing the convergents gives $0, 1, 1/2, 2/3, 5/8$. Looking at the former and latter computed convergents, we note that the third convergent of the latter correctly gives $r' = 3$ while the convergents of the former do not give the correct order when tested using $a^{r'} \bmod N = 1$.

[1] Héctor Abraham *et al.* Qiskit: An Open-source Framework for Quantum Computing. *Available at 10.5281/zenodo.2562110 (2019)*

[2] Smolin, J. A., Gambetta, J. M. & Smith, G. Efficient Method for Computing the Maximum-Likelihood Quantum State from Measurements with Additive Gaussian Noise. *Phys. Rev. Lett.* **108**, 070502 (2012).

[3] Qiskit Learn quantum computing using Qiskit. *Available at https://qiskit.org/textbook/ch-quantum-hardware/measurement-error-mitigation.html*

[4] Tóth, G. Qubit4matlab v3.0: A program package for quantum information science and quantum optics for matlab. *Comput. Phys. Commun.* **179**, 430-437 (2008).

[5] Nielsen, M. A. & Chuang, I. L. *Quantum Computation and Quantum Information: 10th Anniversary Edition* (Cambridge University Press, USA, 2011), 10th edn.
